# New perspectives in patient education for cardiac surgery using 3D-printing and virtual reality

**DOI:** 10.3389/fcvm.2023.1092007

**Published:** 2023-03-03

**Authors:** Maximilian Grab, Fabian Hundertmark, Nikolaus Thierfelder, Matthew Fairchild, Petra Mela, Christian Hagl, Linda Grefen

**Affiliations:** ^1^ Department of Cardiac Surgery, Ludwig Maximilians University Munich, Munich, Germany; ^2^ Chair of Medical Materials and Implants, Technical University Munich, Munich, Germany; ^3^ vr-on GmbH, Munich, Germany

**Keywords:** cardiac surgery, patient education, 3D-printing, virtual reality, preoperative anxiety

## Abstract

**Background:**

Preoperative anxiety in cardiac surgery can lead to prolonged hospital stays and negative postoperative outcomes. An improved patient education using 3D models may reduce preoperative anxiety and risks associated with it.

**Methods:**

Patient education was performed with standardized paper-based methods (*n* = 34), 3D-printed models (*n* = 34) or virtual reality models (*n* = 31). Anxiety and procedural understanding were evaluated using questionnaires prior to and after the patient education. Additionally, time spent for the education and overall quality were evaluated among further basic characteristics (age, gender, medical expertise, previous non-cardiac surgery and previously informed patients). Included surgeries were coronary artery bypass graft, surgical aortic valve replacement and thoracic aortic aneurysm surgery.

**Results:**

A significant reduction in anxiety measured by Visual Analog Scale was achieved after patient education with virtual reality models (5.00 to 4.32, *Δ*-0.68, *p* < 0.001). Procedural knowledge significantly increased for every group after the patient education while the visualization and satisfaction were best rated for patient education with virtual reality. Patients rated the quality of the patient education using both visualization methods individually [3D and virtual reality (VR) models] higher compared to the control group of conventional paper-sheets (control paper-sheets: 86.32 ± 11.89%, 3D: 94.12 ± 9.25%, *p* < 0.0095, VR: 92.90 ± 11.01%, *p* < 0.0412).

**Conclusion:**

Routine patient education with additional 3D models can significantly improve the patients' satisfaction and reduce subjective preoperative anxiety effectively.

## 1. Introduction

Cardiac surgery constitutes a major event in a patient's life. Many patients suffer from postoperative pain, depression and anxiety (1). Preoperative anxiety, caused by uncertainty regarding the surgical procedure and postoperative care, can negatively influence symptoms, the general perioperative outcome and prolong hospital stays (2). Studies show patients' desire for information about their disease as well as surgical procedures and therefore propose a shared decision-making process for better medical outcomes. The improvement of physician-patient communication is of utmost importance in modern patient care (3, 4). Dealing with preoperative anxiety and optimizing patient education can reduce the complication rate and shorten the hospital stay (5). Patient education is most commonly performed by the physician using pre-printed sheets with the option to sketch out individual anatomical or surgical facts. A study revealed that patients cannot recall 40%–80% of medical information given during patient education. Reasons may be the use of complicated medical terminology by the physician, inhibited attention by the patient caused by anxiety or the medium used for patient education, for example spoken or written (6). Using optimized methods for better visualization and especially the combination of haptic and visual methods may improve the patient education (PE) effectively. Especially cardiac anatomy and pathologies are easier to follow when visualized in form of a 3D-printed model or a 3D VR model.

A better visualization during patient education can additionally improve the patient's understanding and thus reduce preoperative anxiety, as shown by previously performed studies in other medical disciplines (7). Biglino et al. could show that the use of 3D-printed cardiac models in congenital heart disease greatly improved patient understanding (8).

3D-printing is an easy and cost-efficient way for producing surgical models used for patient education. It allows for creating patient-specific models for complicated anatomical structures and has already been used for surgical planning and patient or student education and surgical training (9, 10). Another visualization technology is virtual reality (VR), which is not yet commonly used in medicine. It has mostly been used for surgical planning but can also allow for further visualization during patient education (11, 12).

The aim of this study was the comparative analysis of different PE methods for cardiac surgery and their effect on preoperative anxiety and patient understanding. Patients were either educated with (a) conventional pre-printed standardized paper-based methods, (b) 3D-printed models for the corresponding surgery or (c) the use of 3D virtual reality models.

## 2. Materials and methods

### 2.1. Study design

A monocentric randomized controlled trial was completed from December 2019 to March 2022 at the Department of Cardiac Surgery at the LMU university hospital in Munich, Germany. A total of 99 patients were randomized to three different groups of undergoing patient education: using standardized pre-printed paper-based models (control, *n* = 34), 3D-printed models (*n* = 34) and VR models (*n* = 31). Patients’ informed consent was obtained prior to data acquisition and patient data was pseudoanonymized. This study was ethically cleared by the Ethical Committee of LMU university hospital (project number 19–455 KB). Patients that were ≥ 18 years of age and scheduled for a CABG (coronary artery bypass graft surgery), SAVR (surgical aortic valve replacement) or TAA (thoracic aortic aneurysm repair) or a combination thereof were included in this study. Patients were excluded if they were already educated by a physician of the department prior to completing the questionnaires, if they already underwent cardiac surgery before or were not eligible due to different reasons (language barrier, visual or cognitive impairments, emergencies, unable to use VR, experienced dizziness during PE with VR).

Primary outcome of this study was the reduction of presurgical anxiety, secondary outcomes included procedural understanding and patient satisfaction. Prior to patient education, patients took questionnaires for baseline testing regarding anxiety, procedural understanding and patient characteristics. Procedural understanding and overall satisfaction with the PE were evaluated using a self-developed questionnaire. Questions allowed the patient to score knowledge and satisfaction on a five level Likert scale. Anxiety was evaluated by using the Visual Analog Scale (VAS, 1–10), and the State-Trait-Anxiety-Inventory, consisting of the State-Anxiety-Score (STAI) and the Trait-Anxiety-Score (TAI). In this study we used the German short version of the STAI (13), including 10 statements for the state anxiety and 10 statements for the Trait anxiety of the patient. Scores for each short inventory ranged from 10 to 40, while higher scores were associated with higher anxiety levels. The trait anxiety inventory was collected at least one week after cardiac surgery, since it was hypothesized that impending cardiac surgery could falsify the results if they were collected prior to surgery.

**Figure 1:**
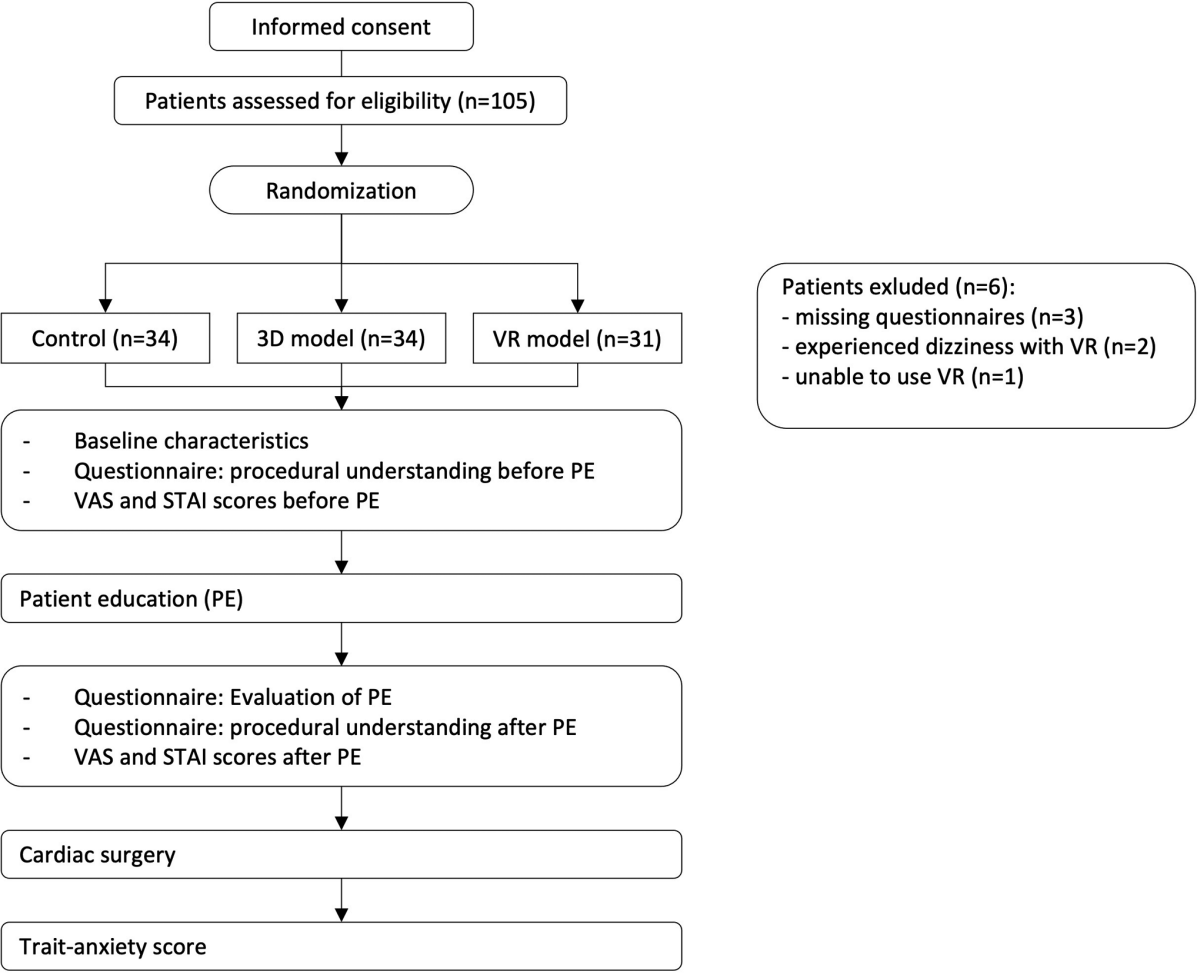
**Overview of study design and randomization**. After informed consent, patients were randomized and completed the described questionnaires at different timepoints.

Patients filled out three questionnaires in total at different time points (Figure 1). The first questionnaire established basic patient characteristics (age, gender, previous knowledge based on medical profession, previous surgery other than cardiac surgery), procedural understanding before patient education and the basic anxiety using VAS and the state anxiety inventory. Subsequently, patients were randomized by starting with the control group and continuing with the 3D model group after reaching the desired number of patients and so on. They were educated according to their group with either standardized pre-printed paper-based models, 3D-printed models or 3D VR models. To prevent investigator's bias the education was performed by the same physician. Following patient education, patients immediately filled out the second questionnaire including questions regarding procedural understanding, anxiety with VAS and state anxiety and questions regarding the quality of the patient education and patients' satisfaction with the corresponding PE method. At least one week after the surgery or at discharge of the patient, the patients were asked to fill out the third questionnaire collecting data regarding the trait anxiety.

### 2.2. Digital model creation

To create the digital models, anonymized contrast-enhanced CT datasets from a preoperative CABG, SAVR and TAA case were imported in Mimics Innovation Suite (Mimics 24.0, Materialise NV, Leuven, Belgium). For all models, a whole-heart model with aortic arch and supra-aortic branches was segmented, displaying the preoperative state of the cardiovascular pathology. After consulting cardiac surgeons, the base model was modified to fit important steps in the procedure. Briefly, the subsequent CABG model included a longer portion of the supra-aortic vessels to show the internal mammary artery. The SAVR model included a cross-section of the left heart without mitral and aortic valve. For the TAA model, isolated versions of the aortic arch were created, displaying a healthy and pathologically enlarged state of the ascending aorta. Furthermore, a model was created removing the ascending aorta distal to the aortic root and proximal to the supra-aortic vessels, preparing for a subsequent implantation of a surgical graft. After designing the individual steps of each procedure, the workflow was divided into the digital pathway towards 3D-printing and the VR application.

### 2.3. 3D-printing

**Figure 2:**
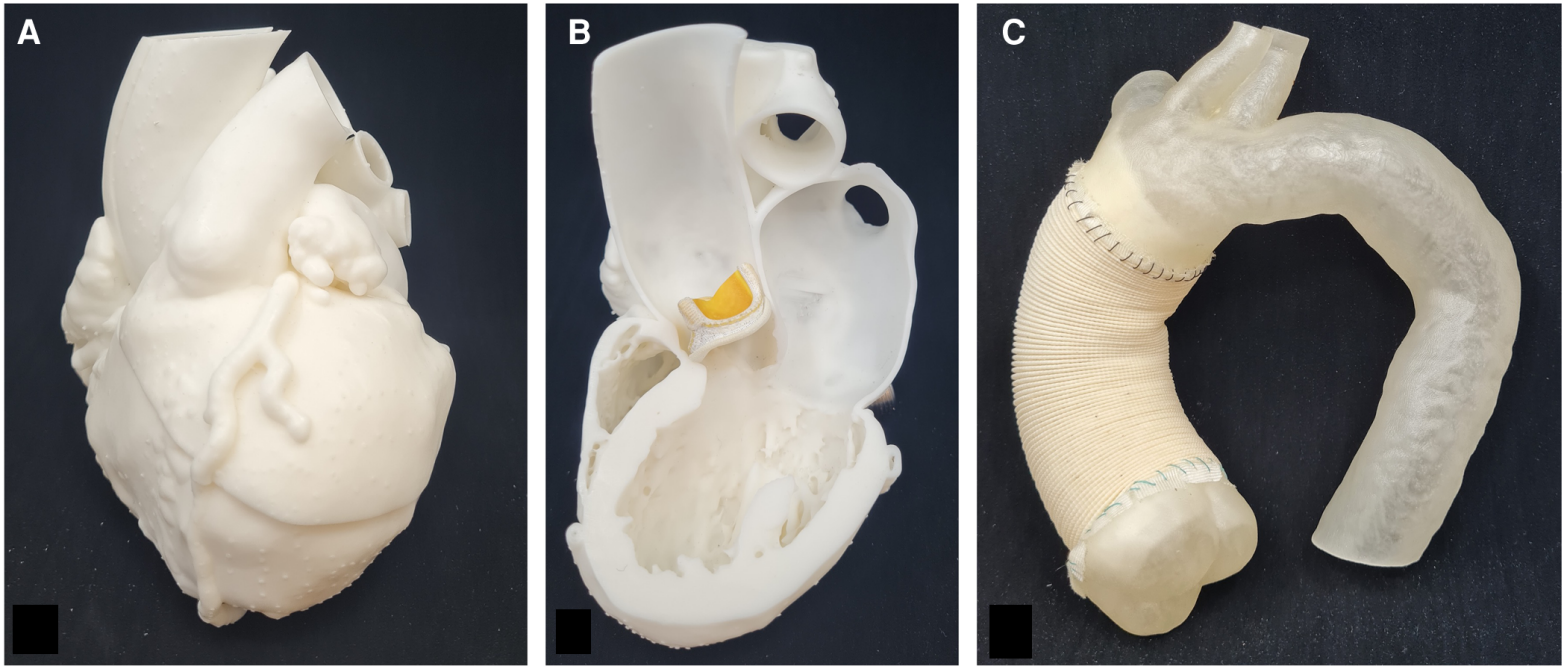
**3D-printed models for CABG, SAVR and TAA**. (**A**) 3D-printed model for CABG surgery to explain anatomy of coronary arteries and anastomosis of bypass grafts. (**B**) Cross-section of the left ventricle for visualization of heart valve placement. In this example, a biological heart valve prosthesis was used for better demonstration. (**C**) 3D-printed flexible model of the aortic branch with a commercial surgical aortic graft sutured to the model for demonstration of repair of the ascending aorta.

To create the 3D-printed models, all digitally created models were exported in the.stl format to transform them into printable files. Files were imported in the respective slicing software depending on the printer type. Whole heart models and cross-sections of the left ventricle were printed using a rigid white material (White_v4, Formlabs Inc., Somerville, MA, USA) on a Formlabs Form 3 (Formlabs Inc.) (Figures 2A,B). Layer height was set to the most precise setting (0.05 mm). After printing, models were post processed according to manufacturer's instructions. For the models of the aortic arch, a flexible printing material (AR-G1L, Keyence Co., Osaka, Japan) was selected and printed using a commercial Polyjet printer (Agilista 3200W, Keyence Co.). A commercial surgical aortic graft (28 mm Gelweave, Vascutek Ltd., Renfrewshire, UK) was sutured to the open model, to create a realistic version of the repaired aorta (Figure 2C). A mechanical heart valve (21 mm On-X, CryoLife Inc, Kennesaw, GA, USA) and a plastic version of a biological heart valve were used as props to explain the SAVR procedure (Figure 2B).

### 2.4. Virtual reality setup

**Figure 3:**
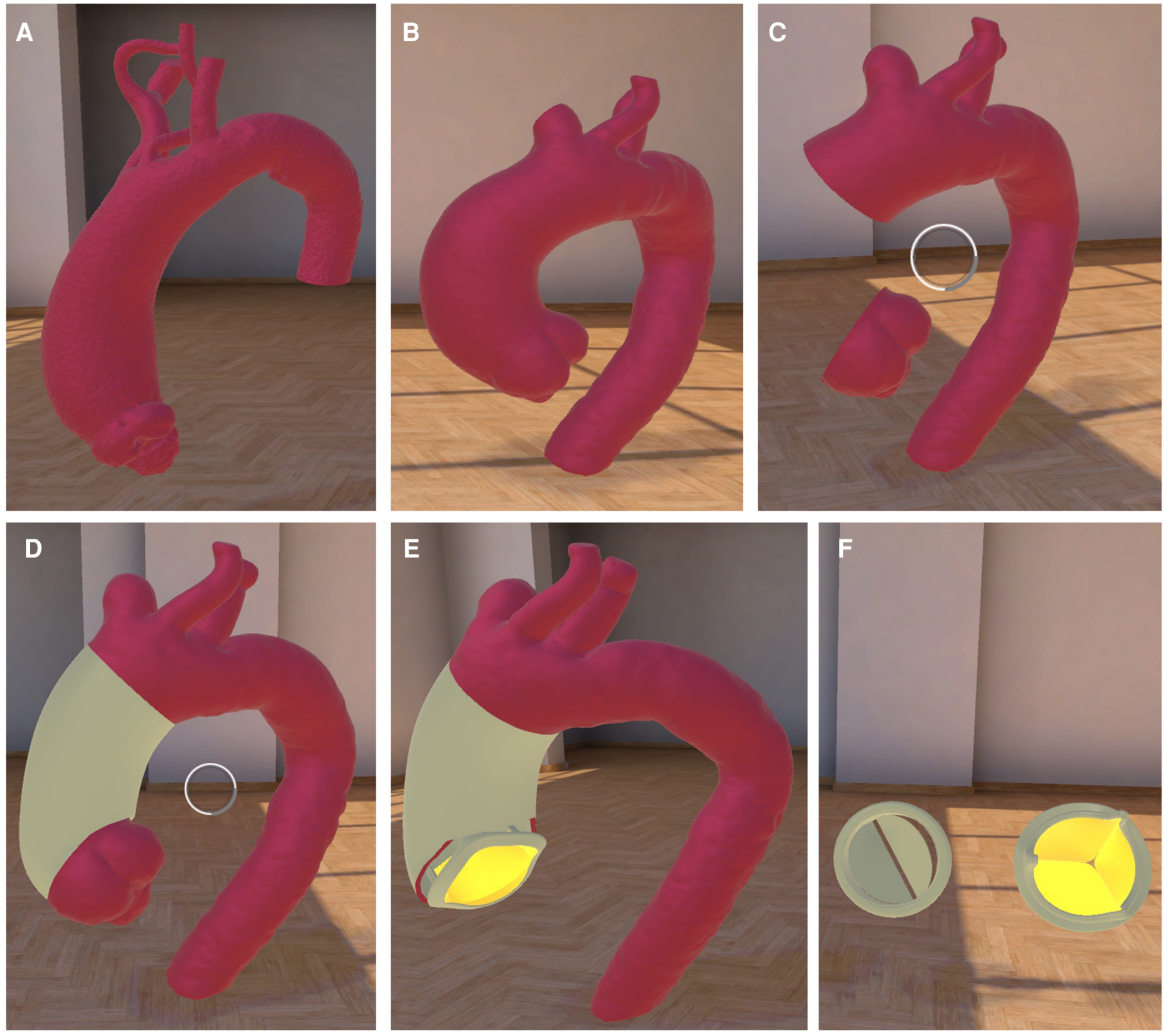
**VR application for TAA**. The VR environment is shown with the example of (**A**) a healthy and normal-sized ascending aorta with the aortic branch. (**B**) showing TAA with simplified surgical steps (**C**) of removing aneurysm and repair of TAA using a surgical aortic graft (**D**). (**E**) displaying the option of additional aortic valve replacement with the option to choose between (**F**) mechanical or biological valve. Round structure in image (**C,D**) shows the pointer.

For the virtual reality application, CABG and TAA models were modified to include surgical grafts at the respective surgical site. For the SAVR models, dummy models of a mechanical and biological heart valve were created. All models were imported in the open source graphic software Blender (14) to align their location in a centralized coordinate system. Furthermore, a collision model was added to the models to allow manipulation in the VR environment. Afterwards, models were imported into Unity (Unity Technologies Inc., San Francisco, CA, USA) to place them in the according presentation and add individual functionality. In cooperation with the startup vr-on (vr-on GmbH, Munich Germany) a custom VR-application based on the company's “STAGE” program, which allows parallel multi-user access, was developed. The app was accessible through both a standard desktop PC and virtual reality goggles (Oculus Quest 2, Meta Platforms Inc., Menlo Park, CA, USA). The application allowed the selection of a specific surgical procedure (CABG, SAVR and TAA) subsequently loading the respective presentation to the user's view (Figure 3 and supplementary material). In the presentation, all participants are placed in a neutral room looking at an upscaled model of the heart. Base functionality for all users was the integration of a laserpointer-style marker (visible for all users), a microphone for communication and free-movement inside the VR room. The moving and exchanging of the individual models was limited to the moderator (i.e., the VR-trained physician) to simplify the handling for novice VR users. The moderator had an advanced interface, allowing to switch between individual surgical steps (Figures 3C,D), showing/hiding implants (Figures 3E,F) and scaling the displayed model.

### 2.5. Statistical analysis

Statistical analysis was performed using GraphPad Prism software (v.9.3.1, GraphPad Software, LLC, La Jolla, CA, USA) and SPSS (v.29, IBM SPSS Statistics for Macintosh, IBM Corp., Armonk, NY, USA). Categorical variables are presented as percentage of the total. Two-Way ANOVA was used for analysis following Šídák's multiple comparisons test after testing for normality with Shapiro-Wilk normality test. Multiple linear regression was performed to assess factors contributing to anxiety and to an increase in procedural understanding. *P* values < 0.05 were considered statistically significant.

## 3. Results

Considering baseline characteristics (Table 1), mean patient age was 64.8 ± 10.9 years, 87% of patients were male, 76.7% previously had non-cardiac surgery and 62.6% designated themselves as previously prepared and informed about the surgery. Baseline scores of STAI and VAS did not differ significantly between the three groups. The most time was needed for the patient education within the VR model group (21.6 ± 3.6 min), whereas PE was performed fastest using pre-printed paper-sheets within the control group (19.6 ± 3.8 min). The Trait-Anxiety-Score showed no significant differences between the groups (control: 21.30 ± 5.32, 3D: 20.82. ± 5.18, VR: 20.39 ± 5.93, *p* = 0.7638).

**Table 1:** Baseline characteristics.

Characteristic	Control (*n* = 34)	3D-Model (*n* = 34)	VR-Model (*n* = 31)	Total (*n* = 99)
CABG	13 (38.24%)	11 (32.35%)	12 (38.71%)	36 (36.40%)
SAVR	11 (32.35%)	13 (38.24%)	10 (32.26%)	34 (34.34%)
TAA	10 (29.41%)	10 (29.41%)	9 (29.03%)	29 (29.30%)
Mean age (SD)	62.94 (13.94)	66.15 (10.06)	65.97 (8.02)	64.86 (10.90)
Gender male	32 (94.12%)	29 (85.29%)	26 (83.87%)	87 (87.87%)
Gender female	2 (5.88%)	5 (14.71%)	5 (16.13%)	12(12.12%)
Medical expertise	5 (14.71%)	2 (6.25%)	3 (9.68%)	10 (10.10%)
Previous non-cardiac surgery	26 (76.47%)	26 (76.47%)	24 (77.42%)	76 (76.76%)
Previously informed	24 (70.56%)	17 (50.00%)	21 (67.74%)	62 (62.62%)
Time for PE (SD)	19.59 (3.81)	20.29 (3.15)	21.61 (3.59)	20.53 (3.56)
STAI baseline (SD)	26.88 (4.80)	24.59 (5.54)	24.32 (6.57)	25.25 (5.68)
Trait-Anxiety-Score (SD)	21.30 (5.32)	20.82 (5.18)	20.39 (5.93)	20.47 (5.42)
Anxiety VAS baseline (SD)	5.38 (2.22)	5.47 (2.67)	5.00 (2.80)	5.48 (2.92)

Baseline characteristics of patients, data shown as percentages or mean ± standard deviation (SD) as shown below.

**Figure 4:**
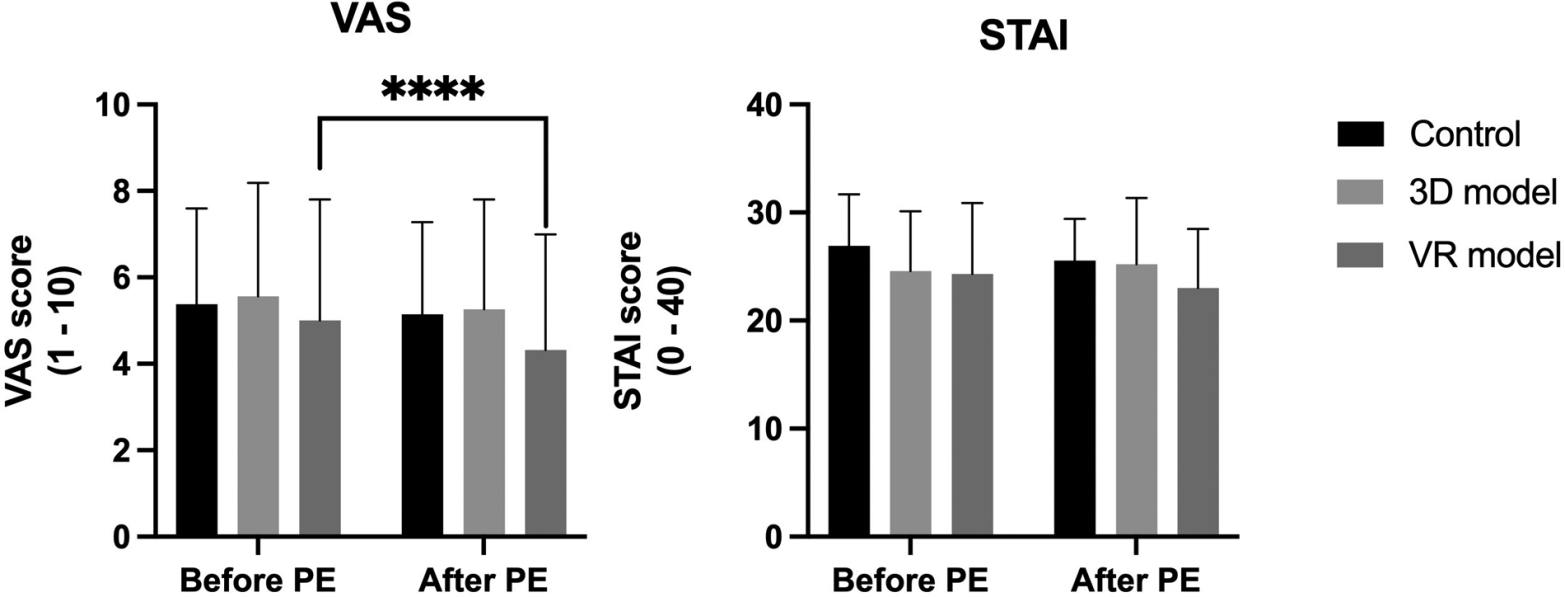
**VAS and STAI anxiety scores before and after PE**. Data represented as mean + standard deviation. ****** **=** ***p*** **<** **0.0001.

The control and 3D model group showed an only slight reduction in anxiety measured with VAS after PE (control: 5.38 to 5.12, *Δ*-0.26 and 3D model: 5.56 to 5.26, *Δ*-0.30). The STAI score after PE with paper sheets was also reduced to some extend (control: 26.91 to 25.56, *Δ*-1.35) whereas the score increased slightly after PE with the 3D-printed models (24.59 to 25.21, *Δ* + 0.62). Results revealed a significant decrease in VAS anxiety for patients educated using VR models (5.00 to 4.32, *Δ*-0.68, *p* < 0.0001) and a reduced, yet not statistically significant, STAI (24.32 to 23.00, *Δ*-1.32) (Figure 4).

**Figure 5:**
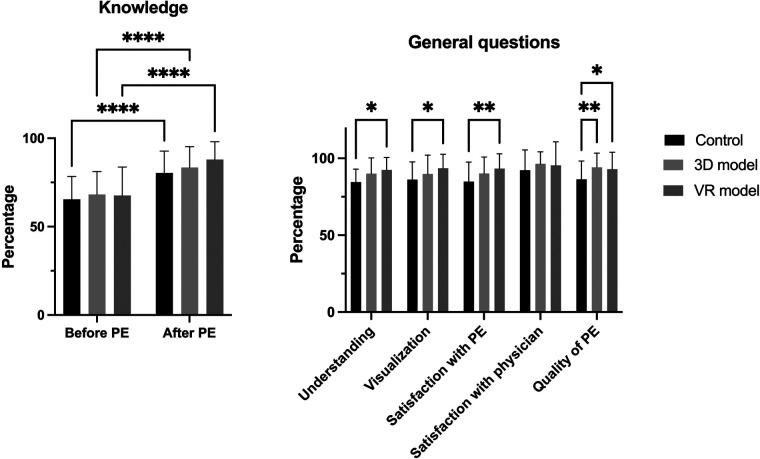
**Knowledge before and after PE; results of general questions regarding PE**. Left: patients’ procedural knowledge before and after PE. Right: results of general questions about understanding, visualization, satisfaction with PE and physician and quality of PE regarding the three study groups. Data represented as mean ± standard deviation. *** **=** ***p*** **<** **0.05, **** **=** ***p*** **<** **0.005, ****** **=** ***p*** **<** **0.0001.

Analysis of procedural knowledge (Figure 5, left graph) revealed a highly statistically significant increase in each group after PE (control: 65.44% to 80.36%, *p* < 0.0001, 3D model: 68.17% to 83.46%, *p* < 0.0001, VR model: 67.62% to 87.98%, *p* < 0.0001). Results for understanding of the surgical procedure (Figure 5, right graph) showed better results after PE with VR (control: 84.60 ± 8.26% and VR model: 92.42 ± 8.15%, *p* = 0.011), as well as a better visualization (control: 86.10 ± 11.53% compared to VR: 93.55 ± 9.15%, *p* < 0.017). Patients were more satisfied with PE using 3D models and VR rather than paper-sheets (control: 84.80 ± 12.74% compared to VR: 93.33 ± 9.58%, *p* < 0.0038). There were no statistically significant differences in the patients' satisfaction with the physician performing the PE. Patients rated the quality of PE using both visualization methods (3D and VR models) higher compared to conventional paper-based methods (control: 86.32 ± 11.89%, 3D: 94.12 ± 9.25%, *p* < 0.0095, VR: 92.90 ± 11.01%, *p* < 0.0412).

Multiple linear regression was used to analyze contributing factors to anxiety levels prior to the PE (VAS1 and STAI1, Table 2) and after PE (VAS2 and STAI2). Analysis revealed statistically significant results for VAS1 [*R*^2 ^= 0.451, *F* (8, 90) = 9.227, *p* < 0.001]. Every increase of STAI1 score lead to a significant increase in VAS1 score (*B* = 0.367, *p* < 0.001). A higher score on the questionnaire 1 regarding procedural knowledge before the PE lead to a decreased VAS1 score (*B* = −0.197, *p* = 0.001). Patients with previous medical expertise or knowledge show a significant increase in STAI1 score (*B* = 2.293, *p* = 0.029). Other tested variables, as shown in Table 2, did not contribute significantly to a change in anxiety scores before the PE. When analyzing contributing factors to the corresponding anxiety scores after the PE (VAS2 and STAI2) with the same variables, results revealed a significant decrease in VAS1 [*R*^2^ = 0.324, F (9, 89) = 6.227, *p* < 0.001] score when patients were previously informed about the impending procedure (B = −1,39, *p* = 0.003).

**Table 2:** Analysis of anxiety scores before the PE.

Model		*B*	SE	*β*	*p*
1^a^	(Intercept)	2.333	3.015		0.441
	Age	0.028	0.019	−0.117	0.155
	Gender	−0.318	0.634	−0.041	0.617
	Medical expertise	0.064	0.796	0.007	0.936
	Previous surgery	0.402	0.496	0.068	0.420
	Previously informed	−0.676	0.486	−0.131	0.168
	Questionnaire 1 score	−0.197	0.060	−0.321	**0**.**001**
	STAI1	0.367	0.069	0.455	**<.001**
	TAI	0.022	0.048	0.037	0.648
2^b^	(Intercept)	19.709	3.463		<.001
	Age	−0.012	0.026	−0.041	0.644
	Gender	−0.827	0.843	−0.086	0.329
	Medical expertise	2.293	1.036	0.199	**0**.**029**
	Previous surgery	−0.230	0.664	−0.031	0.730
	Previously informed	−0.093	0.656	−0.014	0.888
	Questionnaire 1 score	0.032	0.085	0.043	0.703
	TAI	0.090	0.064	0.123	0.158
	VAS1	0.654	0.123	0.527	**<.001**

Analysis of contributing factors. Gender defined as 0 = female, 1 = male. *B* = unstandardized coefficients, *SE = *standard error, *β =*^ ^standardized coefficients. VAS1, STAI1 questionnaire 1 scores were collected prior to PE.

^a^ dependent Variable: VAS1.

^b^ dependent Variable: STAI1.

Regression analysis of patients' procedural knowledge (Table 3) before the PE measured with questionnaire 1 showed statistically significant results [*R*^2^ = 0.380, *F*(8, 90), *p* < 0.001]. Patients that underwent any other surgery than cardiac surgery before achieved lower scores when filling out questionnaire 1 regarding knowledge about heart disease and the planned procedure (*B* = −1.966, *p* = 0.016). The score was also lower for an increased VAS1 score (*B* = −0.543, *p* = 0.001). Patients that were previously informed show significantly higher scores on the questionnaire 1 results (B =  + 3.018, *p* < 0.001). The only statistically significant contributing factor to an increased score on the second questionnaire [*R*^2^ = 0.262, *F*(10,88) = 4.477, *p* < 0.001] after the PE was the overall quality of the PE (*B* =  + 0.261, *p* < 0.001). Other variables tested did not reveal significant contribution.

**Table 3:** Analysis of procedural knowledge before and after the PE measured with questionnaire at timepoint 1 and 2.

Model		*B*	SE	*β*	*p*
1^a^	(Intercept)	27.506	4.104		<.001
	Age	−0.058	0.032	−0.151	0.069
	Gender	−1.486	1.043	−0.117	0.158
	Medical expertise	1.975	1.307	0.130	0.134
	Previous surgery	−1.966	0.801	−0.204	**0**.**016**
	Previously informed	3.018	0.751	0.359	**<.001**
	TAI	0.041	0.080	0.042	0.608
	STAI1	0.050	0.131	0.038	0.703
	VAS1	−0.543	0.165	−0.333	**0**.**001**
2^b^	(Intercept)	8.559	5.139		0.099
	Age	−0.018	0.028	−0.060	0.508
	Gender	0.233	0.904	0.023	0.797
	Medical expertise	1.238	1.110	0.103	0.268
	Previous surgery	−0.640	0.693	−0.084	0.359
	Previously informed	1.071	0.655	0.161	0.106
	TAI	0.018	0.068	0.024	0.790
	Time	0.056	0.084	0.061	0.506
	STAI2	0.100	0.131	0.078	0.446
	Quality of PE	0.261	0.062	0.402	<.001
	VAS2	−0.255	0.146	−0.191	0.083

Analysis of contributing factors. Gender defined as 0 = female, 1 = male. *B* = unstandardized coefficients, *SE = *standard error, *β =*^ ^standardized coefficients. VAS1 and STAI1 scores were collected prior to PE, whereas VAS2 and STAI2 were collected after PE.

^a^ dependent Variable: questionnaire 1.

^b^ dependent Variable: questionnaire 2.

## 4. Discussion

Patients undergoing cardiac surgery can suffer from a high level of perioperative anxiety, especially regarding the lack of information or knowledge about the impending surgery (2). Williams et al. could show that preoperative anxiety provides a higher risk of in-hospital mortality in patients undergoing cardiac surgery (15). An attempt to decrease patients' anxiety is either pharmacological, using anxiolytic medication prior to surgery, or non-pharmacological, e.g., optimizing patient education to increase patients' procedural understanding (16). The latter can be achieved by multiple different approaches, especially optimizing patient-physician communication and visualization of anatomical structures and surgical steps.

In this study, we initially established a workflow to create 3D-printed and VR models for patient education and clinical application, showing the main steps of the surgical procedure (in this case SAVR, CABG and TAA). Previous studies revealed visual and haptic tools to enhance learning and understanding (17). Our findings are in accordance with these studies; patients were generally satisfied with the use of 3D models and especially welcomed the visual experience using VR for procedural understanding. Results revealed a statistically significant decrease in anxiety measured with the VAS after PE using VR and a slight decrease in STAI after using VR for the PE. There were no noticeable differences after PE using paper-based sheets or 3D-printed models. As for general knowledge, every group benefited greatly regardless of the medium used and there was no difference in satisfaction with the physician performing the PE within the groups. In accordance to the study by Marquess et al. we could show that the use of virtual reality significantly improved the patients' comprehension and procedural understanding (18). Visualization was improved using both 3D-printed and VR models and patients were especially satisfied with the use of virtual reality in a clinical setting, as confirmed by other studies (11). The overall quality of the PE was best rated using 3D models followed by VR models.

The results revealed a noteworthy and significant increase of State-Trait-anxiety score before the PE for patients with medical expertise. Reviewing the current literature did not result in any findings regarding the correlation of medical knowledge/expertise or even the factor of being a medical professional with preoperative anxiety. A study by Gillies et al. evaluating the effect of anaesthetic information prior to surgery regarding anxiety investigated the effect of providing a booklet with information to the patients. While the majority of the patients found the booklet to be helpful, 35% of patients found the information given to be worrisome to some degree (19). It could be hypothesized that having a medical background might lead to higher preoperative anxiety levels because of the detailed knowledge about possible complications. Investigation of this correlation is a future research project of our work group. Further analysis showed that patients that were informed before the PE had significantly lower anxiety scores measured with the VAS and achieved significantly higher scores on the second questionnaire evaluating procedural understanding after the PE. Previous surgery other than cardiac surgery was identified as a factor contributing negatively on the outcome of procedural knowledge evaluated by the first questionnaire. Whereas the score of the second questionnaire collecting data on patients' knowledge after PE correlated with the quality of the PE and was correspondingly higher the better the quality of the PE was rated by the patients.

The heterogeneous collective regarding pathologies and corresponding surgeries (CABG, SAVR, TAA) must be mentioned as a limitation of this study as it can influence anxiety levels. In addition to evaluating the preoperative anxiety, this study was aiming at establishing 3D and VR models for patient education and creating a workflow for the clinical application thereof. The herein presented results allow for a following future research project with a) patient-specific 3D-printed and VR models and b) the evaluation of preoperative anxiety in regard to the specifically planned surgical procedure. Furthermore, we would like to investigate the effect of the corresponding educational methods on postoperative outcomes, e.g., complication rate or duration of hospital stay, in a larger cohort of patients undergoing cardiac surgery in a future research project. This study constitutes a proof-of-concept and establishment of workflows for further studies.

In times of digitalization, 3D-printing and virtual reality are innovative technologies that can improve the experience of patient education greatly and optimize the step-by-step explanation of surgical steps for patients. The clinical translation of these technologies is long overdue. The virtual reality setup allowed for the interaction between the patient and the physician with the 3D models within the virtual reality environment and encouraged an open discussion about procedural steps. Although patients were most satisfied as well as content with the visualization using VR, PE using 3D-printed models was rated best for overall quality. Even though there was no significant difference in time spent performing the PE, the education using VR took approximately 2 min longer and required the previous setup of a laptop and a brief introduction of how to handle the VR controller and glasses prior to the PE. Two patients were excluded for experiencing dizziness while using VR glasses and one patient was excluded for not being able to handle the controllers because of a strong tremor of the hands. Routine implementation of VR technology in a clinical setting might still be prevented by missing infrastructure, for example regarding WIFI access. Additionally, as mentioned above, not every patient is eligible or qualified to be educated using VR. At this point it should also be mentioned that the PE with standardized paper-based models already revealed good results regarding patient knowledge. This fact demonstrates that the conventional method of patient education has its reason for clinical application over the years. The herein presented results suggest that the use of visual and haptic models further improve the patients' educational experience. Considering the availability of additive manufacturing nowadays and the fact that the visualization and quality of PE was also optimized using 3D-printed models, it might be easier to start with a routine implementation of an enhanced PE using 3D-printed models with the option to create patient-specific models for PE in the future. In this regard, the topic of cost effectiveness depends on a variety of factors and is very individual. Of course, creating and printing the 3D models is possible on a low budget, but the purchasing and software licensing for the setup of the 3D printer also have to be taken into account. The same applies for VR goggles and the corresponding software licenses and should therefore be considerer for each use case individually.

## Data Availability

The raw data supporting the conclusions of this article will be made available by the authors, without undue reservation.
