# Supplementary material for: New perspectives in patient education for cardiac surgery using 3D-printing and virtual reality
Source: Front Cardiovasc Med. 2023 Mar 3;10:1092007. doi: 10.3389/fcvm.2023.1092007 (PMC10020687; doi:10.3389/fcvm.2023.1092007)
Supplement: Supplementary file 1 [file Datasheet1.pdf]

Questionnaire 1

Gender	male	female	diverse
Age	years		
Do you have professional knowledge (e.g. job in medicine, biology) about the biology, anatomy and function of the heart?	Yes	No	
Have you had surgery before?	Yes	No	
Have you read up on or informed yourself on your impending surgery?	Yes	No	

Please rate the following sentences	**Strongly disagree**	**Disagree**	**Agree**	**Strongly Agree**	**Neither agree nor disagree**
I have solid knowledge about the anatomy and function of the heart.	○	○	○	○	○
I know the basic anatomy of the cardiovascular system.	○	○	○	○	○
I understand my individual cardiac pathology and disease.	○	○	○	○	○
I know which part of my heart carries the pathology.	○	○	○	○	○
I have a good knowledge about the surgical procedure.	○	○	○	○	○
I want a detailed patient education about the surgical steps.	○	○	○	○	○
I understand the positive effect the surgery will have on my health.	○	○	○	○	○
I'm confident to explain the main steps of the surgical procedure to someone else.	○	○	○	○	○

Please rate your current anxiety level in regard to the surgery on the following scale 1 to 10:

1	2	3	4	5	6	7	8	9	10

1 = No anxiety → 10 = Extreme anxiety

Please give the answer which seems to describe your ***present*** *feeling best.*	**Not at all**	**Somewhat**	**Moderately so**	**Very much so**
I feel calm	○	○	○	○
I am tense	○	○	○	○
I feel at ease	○	○	○	○
I am presently worrying over possible misfortunes	○	○	○	○
I am frightened	○	○	○	○
I feel nervous	○	○	○	○
I am jittery	○	○	○	○
I am relaxed	○	○	○	○
I am worried	○	○	○	○
I feel steady	○	○	○	○
