# Supplementary material for: New perspectives in patient education for cardiac surgery using 3D-printing and virtual reality
Source: Front Cardiovasc Med. 2023 Mar 3;10:1092007. doi: 10.3389/fcvm.2023.1092007 (PMC10020687; doi:10.3389/fcvm.2023.1092007)
Supplement: Supplementary file 2 [file Datasheet2.pdf]

Questionnaire 2

Please rate the following sentences	**Strongly disagree**	**Disagree**	**Agree**	**Strongly Agree**	**Neither agree nor disagree**
I have solid knowledge about the anatomy and function of the heart.	○	○	○	○	○
I know the basic anatomy of the cardiovascular system.	○	○	○	○	○
I understand my individual cardiac pathology and disease.	○	○	○	○	○
I know which part of my heart carries the pathology.	○	○	○	○	○
I have a good knowledge about the surgical procedure.	○	○	○	○	○
I want a detailed patient education about the surgical steps.	○	○	○	○	○
I understand the positive effect the surgery will have on my health.	○	○	○	○	○
I'm confident to explain the main steps of the surgical procedure to someone else.	○	○	○	○	○

Please rate your current anxiety level in regard to the surgery on the following scale 1 to 10:

1	2	3	4	5	6	7	8	9	10

1 = No anxiety → 10 = Extreme anxiety

*Please give the answer which seems to describe your **present** feeling best.*	**Not at all**	**Somewhat**	**Moderately so**	**Very much so**
I feel calm	○	○	○	○
I am tense	○	○	○	○
I feel at ease	○	○	○	○
I am presently worrying over possible misfortunes	○	○	○	○
I am frightened	○	○	○	○
I feel nervous	○	○	○	○
I am jittery	○	○	○	○
I am relaxed	○	○	○	○
I am worried	○	○	○	○
I feel steady	○	○	○	○
