# Supplementary material for: New perspectives in patient education for cardiac surgery using 3D-printing and virtual reality
Source: Front Cardiovasc Med. 2023 Mar 3;10:1092007. doi: 10.3389/fcvm.2023.1092007 (PMC10020687; doi:10.3389/fcvm.2023.1092007)
Supplement: Supplementary file 3 [file Datasheet3.pdf]

Questionnaire 3

*Please give the answer which seems to describe your **general** feeling.*	**Almost never**	**Sometimes**	**Often**	**Almost always**
I feel nervous and restless	○	○	○	○
I feel satisfied with myself	○	○	○	○
I wish I could be as happy as others seem to be	○	○	○	○
I feel like a failure	○	○	○	○
I worry too much over something that really doesn’t matter	○	○	○	○
I lack self-confidence	○	○	○	○
I feel secure	○	○	○	○
I feel inadequate	○	○	○	○
I am a steady person	○	○	○	○
I get in a state of tension or turmoil as I think over my recent concerns and interests	○	○	○	○
