# Supplementary figures and images for: New perspectives in patient education for cardiac surgery using 3D-printing and virtual reality
Source: Front Cardiovasc Med. 2023 Mar 3;10:1092007. doi: 10.3389/fcvm.2023.1092007 (PMC10020687; doi:10.3389/fcvm.2023.1092007)

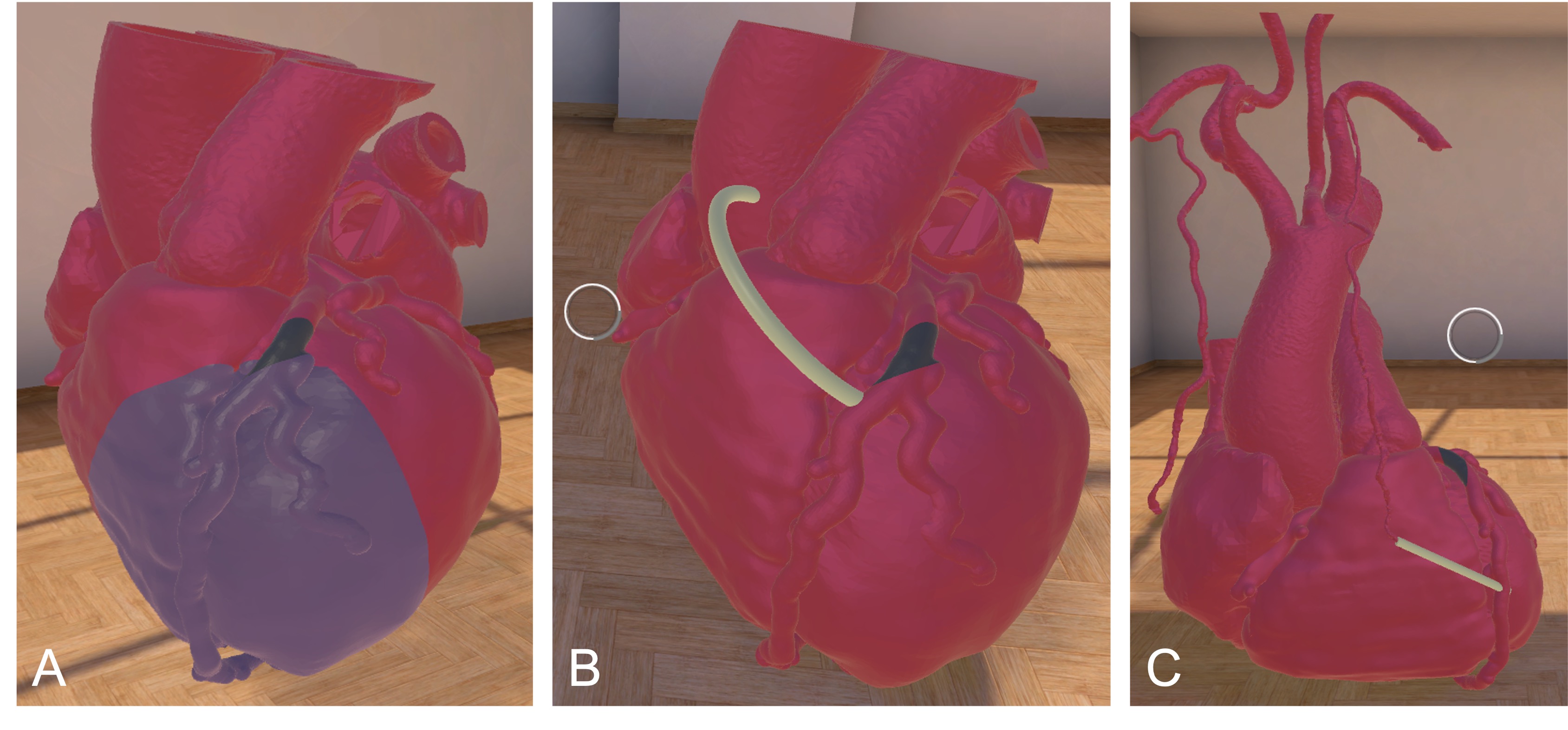

Supplement: Supplementary file 4 [file Image1.jpeg]

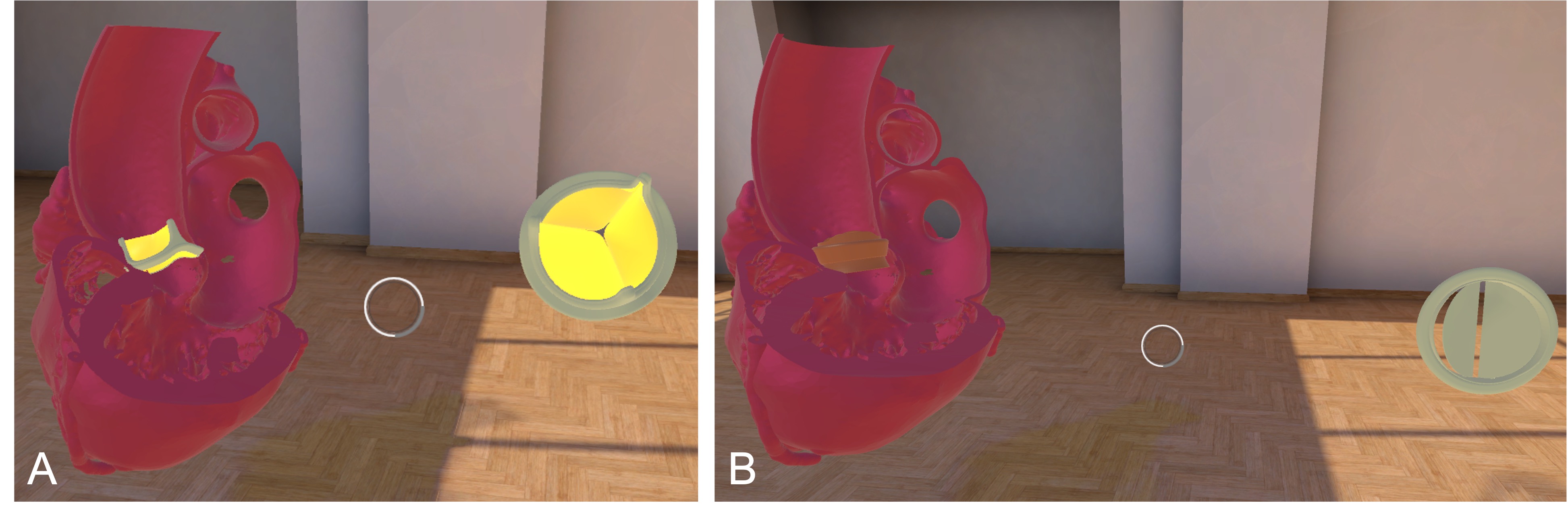

Supplement: Supplementary file 5 [file Image2.jpeg]
